# Estimation of the measurement uncertainty and practical suggestion for the description of the metrological traceability in clinical laboratories

**DOI:** 10.11613/BM.2021.010501

**Published:** 2020-12-15

**Authors:** Raúl Rigo-Bonnin, Noelia Díaz-Troyano, Laura García-Tejada, Albert Marcè-Galindo, Míriam Valbuena-Asensio, Francesca Canalias

**Affiliations:** 1 Laboratori Clínic, IDIBELL, Hospital Universitari de Bellvitge, L’Hospitalet de Llobregat, Barcelona, Spain; 2 Laboratori de Referència d’Enzimologia Clínica, Departament de Bioquímica i Biologia Molecular, Universitat Autònoma de Barcelona, Bellaterra, Spain

**Keywords:** biostatistics, quality control, standardization, traceability, uncertainty

## Abstract

Clinicians request a large part of measurements of biological quantities that clinical laboratories perform for diagnostic, prognostic or diseases monitoring purposes. Thus, laboratories need to provide patient’s results as reliable as possible. Metrological concepts like measurement uncertainty and metrological traceability allow to know the accuracy of these results and guarantee their comparability over time and space. Such is the importance of these two parameters that the estimation of measurement uncertainty and the knowledge of metrological traceability is required for clinical laboratories accredited by ISO 15189:2012. Despite there are many publications or guidelines to estimate the measurement uncertainty in clinical laboratories, it is not entirely clear what information and which formulae they should use to calculate it. On the other hand, unfortunately, there are a small number of clinical laboratories that know and describe the metrological traceability of their results, even though they are aware of the lack of comparability that currently exists for patient’s results. Thus, to try to facilitate the task of clinical laboratories, this review aims to provide a proposal to estimate the measurement uncertainty. Also, different suggestions are shown to describe the metrological traceability. Measurement uncertainty estimation is partially based on the ISO/TS 20914:2019 guideline, and the metrological traceability described using the ISO 17511:2020. Different biological quantities routinely measured in clinical laboratories are used to exemplify the proposal and suggestions.

## Introduction

The measured values of biological quantities facilitated by clinical laboratories provide essential information that conditions the correct clinical orientation, optimisation of patients’ healthcare process, and lead to appropriate therapeutic, diagnostic, or healthcare actions. Therefore, these values must be reliable (exact) and comparable with other ones obtained in different periods and places (traceable) (*1*, *2*).

Metrological concepts like measurement uncertainty (MU) and metrological traceability (MT) allow to know the degree of accuracy of the measured values that a clinical laboratory provides, and the comparability or transferability of these results over time and space. Currently, such is the importance of these two concepts that the estimation of MU and the knowledge of MT are required for clinical laboratories accredited by ISO 15189:2012 (*3*).

Measurement uncertainty complements a measured value of a biological quantity, indicating the magnitude of the doubt about this value and providing a quantitative indication of its quality and reliability (*4*). Nowadays, there are two main approaches for estimating MU: so-called *bottom-up* and *top-down*. The *bottom-up* approach is based on a comprehensive categorisation of the measurement where each potential uncertainty source is identified and quantified. The estimates of uncertainty expressed as standard deviations (standard uncertainties) are assigned to individual components of the procedure, which are then mathematically combined using propagation rules to provide a combined standard uncertainty. Finally, an expanded uncertainty is estimated, multiplying the combined uncertainty by an appropriate coverage factor (*1*).

Conversely, the *top-down* approach considers uncertainty as a whole. First, the most significant uncertainty sources are identified and grouped. Then, their standard uncertainties are estimated using available laboratory tests performance information, such as measurement procedure validation or verification data, and intra-laboratory or inter-laboratory data (*e.g.* internal and external quality control data). Subsequently, the combined uncertainty is obtained from the standard uncertainties for, finally, to estimate the expanded uncertainty (*1*).

Furthermore, MT is defined as the property of a measurement result whereby the result can be related to a reference through a documented unbroken chain of calibrations, each contributing to the measurement uncertainty (*4*). In other words, to achieve comparability of results over space and time, it is essential to link all the individual measurement results to some common reference. In this way, results can be compared through their relationship to that reference. Ideally, this reference should be an International System (SI) unit of measurement materialised by a primary reference measurement procedure and a primary measurement standard (*5*).

At present, there are several guidelines and publications that clinical laboratories could use to estimate the MU, but there is still no consensus on how they should calculate the MU (*6*-*18*). On the contrary, there does seem to be an agreement on how laboratories should describe the MT of the measurement results they provide. Nevertheless, there are a small number of clinical laboratories that know the MT of their results, even though they are aware of the lack of comparability that currently exists for patient’s results (*13*, *17*-*19*). Thus, with the intention to facilitate the task of clinical laboratories, this review aims to provide a proposal to estimate the measurement uncertainty in clinical laboratories showing examples to decide what information and which formulae they should use to calculate the MU. Also, practical suggestions are provided to enable laboratories to describe the MT of their results.

## Measurement uncertainty estimation

The *top-down* approach is particularly well suited to measuring systems commonly encountered in clinical laboratories. So, the MU should be estimated using this approach and taking into account the following steps (*6*):

### Specification of the measurand

A measurand is defined as the quantity intended to be measured (*4*). So, the measurand must be unequivocally defined and the measurement procedure used must be exhaustively detailed; otherwise, an insufficient specification of the measurand may itself be a significant uncertainty source (definitional or intrinsic uncertainty) that could be difficult to estimate. To specify the measurand, it is necessary to include at least the following information (*6*, *20*):

The name of the biological system containing the component (analyte), *e.g.* blood, plasma, serum, urine, *etc.*
The name of the biological component (so-called analyte), *e.g.* glucose, sodium ion, sirolimus, troponin T, *etc.*
The kind-of-property, *e.g.*, substance concentration, mass concentration, number concentration, catalytic concentration, *etc.*
The measurement unit, *e.g.*, mmol/L, mg/L, entities/L, µkat/L, *etc.*

Sometimes, it is also necessary to include additional information such as the measuring system (or the measurement method or the measurement principle) used to measure the quantity, and the conditions under which the measurements are performed (*e.g.*, the temperature for enzymes).

### Identification of the uncertainty sources

According to different clinical laboratory guidelines, the most significant uncertainty sources contributions to the overall MU are captured by the uncertainties related to the assigned value of the end-user calibrator (*u*_cal_), the long-term intermediate precision (*u*_Rw_), and the bias (*u*_b_) (*6*, *7*). Thus, it would be sufficient that clinical laboratories consider only these three uncertainty sources to provide reasonable estimates of MU that help ensure that patient results are fit for medical use.

### Estimation of the standard uncertainties

#### Uncertainty related to the assigned value of the end-user calibrator

A correct estimate of MU is indeed not possible without the *u*_cal_ because it includes all uncertainties contributions accumulated across the entire traceability chain of a measurement result (*13*). Thus, clinical laboratories should include the *u*_cal_ in the uncertainty budget when they estimate the MU. The *in vitro* diagnostic (IVD) manufacturers are requested to comply with the European Regulation 2017/746 on *in vitro* medical diagnostics and must be provided with this information to clinical laboratories (*21*). Usually, manufacturers present this information as the calibration material assigned value (x_cal_) jointly with its expanded uncertainty (*U*_cal_ or %*U*_rel(cal)_) using a coverage factor (*k*) equal to 2. So, the *u*_cal_ can be obtained as:


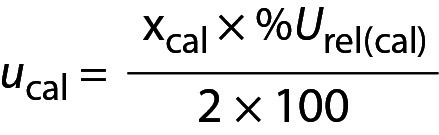


or


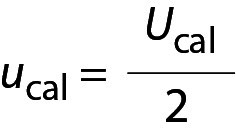


Instead, when clinical laboratories prepare their calibration materials, they are entirely responsible for estimating the *u*_cal_. In these cases, the *u*_cal_ can be calculated taking into account all information used to prepare the calibration materials, and statistically combining the uncertainties associated with each one of the sequential value assignment steps utilising the *law for the propagation of uncertainty* (*8*, *9*, *22*).

#### Uncertainty related to the long-term intermediate imprecision

Most of the components of the MU are included in the long-term intermediate imprecision. This imprecision can be calculated from internal quality control (IQC) data (*6*).

When clinical laboratories estimate the *u*_Rw_, there are different considerations that they should take into account (*6*, *7*, *13*):

- The IQC materials used for estimating the *u*_Rw_ should comply with specific attributes or characteristics. For example, the materials should be commutable and different from that used to check the correct alignment of the measuring systems.
- The IQC material data must be collected for a sufficiently long-time-interval to reflect most of the sources of variability influencing the measurement process.
- Different IQC material levels at mean values close to important medical decision limits should be used to know the *u*_Rw_ behaviour across the measuring interval of the measuring systems.
- A precision study (*e.g.,* comparing variances using the *F*-test) of representative human samples and IQC materials should be performed to verify that the magnitude of imprecision for both materials is similar. An example of how to assess this type of studies was published by Fuentes-Arderiu *et al*. (*23*).
- In clinical laboratories, it is common to indistinctly measure a biological quantity with more than one identical measuring system (or different modules of the same measuring system). Therefore, it would be advisable to obtain an estimate of MU that would have the variation overall measuring systems.
- To avoid the effect of IQC material lot changes on estimating uncertainty, as well as for practical reasons, the use of a single IQC material lot during the estimation study it would be advisable (*6*, *8*).

As far as possible, clinical laboratories should comply with most of these considerations to perform an adequate *u*_Rw_ estimation, as well as to avoid a possible over-estimate of the *u*_Rw_.

When only one calibrator lot, IQC lot, and a unique measuring system are used during a specified time interval, the *u*_Rw_ can be calculated as the classical standard deviation (s):


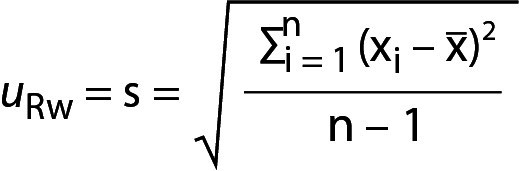


where x_i_ represents IQC values obtained in a specified time-interval, n the number of IQC replicate measurements in a specified time-interval and x the IQC mean value obtained in a specified time-interval.

Furthermore, when two or more lots of calibration or IQC materials are involved in a specified time interval, or when two or more identical measuring systems are used to measure the same biological quantity, the *u*_Rw_ can be calculated as a pooled standard deviation (s_p_) (*22*):


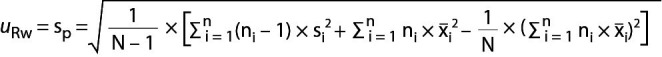


with a pooled mean (x_p_) given by:


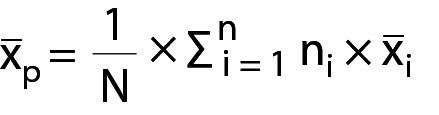


where n_i_ is the number of IQC replicate measurements using the calibrator lot i (or the number of IQC replicate measurements using the IQC lot i; or the number of IQC replicate measurements using measuring system i), s_i_ is the standard deviation obtained using the calibrator lot i (or the standard deviation obtained using the IQC lot i, or the standard deviation obtained using the measuring system i), x_i_ is the IQC mean value obtained using the calibrator lot i (or the IQC mean value obtained using the IQC lot i, or the IQC mean value obtained using the measuring system i), x_p_ is the IQC pooled mean calculated using all calibrator lots (or IQC pooled mean calculated using all IQC lots, or IQC mean value calculated using all measuring systems) and N is the total number of IQC replicate measurements.

#### Uncertainty related to the bias

At present, how to deal with the bias on clinical measurements and how to calculate the bias component of uncertainty continues to be a matter of debate. Some authors firmly state that the bias (or its uncertainty) must not be included in the uncertainty budget because the bias component is already will be part of the *u*_cal_ (*13*). In other words, it is expected that IVD manufacturers must ensure the traceability of their measuring systems to the highest-order available references. This statement is partially correct because it is known that, in several cases, the IVD manufacturers continue to prepare their calibration materials *in-house* without any traceability to high-order metrological references, although they are requested to comply with European Regulation 2017/746.

In contrast, other authors opine that when a significant bias is detected this one should be eliminated (*24*, *25*). If the bias cannot be eliminated, there are two ways of proceeding: 1) to correct the bias by applying a correction factor and incorporating its uncertainty to the uncertainty budget, or 2) to include the bias itself in the uncertainty budget. It should be noted that the first point would only be applicable for those cases in which the bias study is assessed using certified reference material (CRM), and when the traceability declared by the IVD manufacturer is to the same CRM used to evaluate the bias study (*24*, *25*).

Regarding the *u*_b_, different procedures allow estimating the measuring system bias, is the one based on the use of reference materials the most widely used. Reference material can be a CRM, an IQC material (with or without an associated IQC inter-laboratory scheme), or a control material belonging to an external quality assurance service (EQAS) (*6*, *7*, *25*). Of all of them, CRM or commutable IQC or EQAS control materials with values assigned by an international conventional or primary measurement procedures should be used whenever possible. In the absence of these CRM or commutable control materials, inter-laboratory IQC followed by control materials from an EQAS can be used (*14*).

When a CRM is used to estimate the bias (b), the b and its uncertainty (*u*_b_) can be calculated as (*24*):


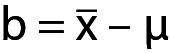


*u*_b_ = *u*_μ_ (Eq. 7),

where x designates the mean value obtained after processing the CRM in a specific time-interval, μ is the CRM assigned value and *u*_μ_ the uncertainty associated with the CRM assigned value. Note that Eq. 5 should be used to calculate the x value if more than one calibration lot or measuring system is used.

Bias studies using IQC materials can be performed following the Farrance *et al.* recommendations (*22*):

When two or more lots of calibrator or IQC materials are involved in a specified time interval, or when two or more identical measuring systems are used to measure the same biological quantity, the bias can be calculated as a weighted mean value of bias (b_w_) (*24*):
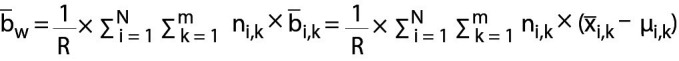

and its uncertainty () as:


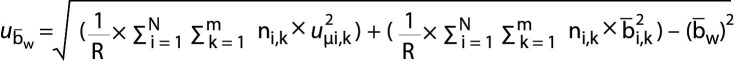


The ICQ manufacturer must provide the *μ*_i,k_. Otherwise, if the IQC material presents an associated IQC inter-laboratory scheme, it can be estimated as (*26*):


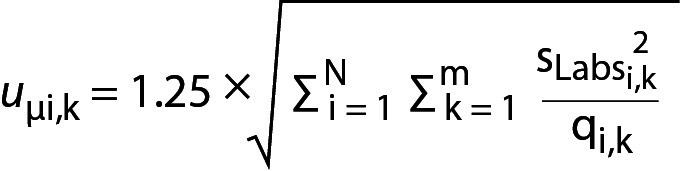


In the previous equations R represents the total pool size (total number of replicate measurements, *i.e.*, of IQC values), N the number of IQC materials levels used, m the number of calibrator lots (or IQC material lots or measuring systems) used, n_i,k_ the number of replicate measurements using the IQC material level i for the calibrator lot k (or IQC material k, or measuring system k), b_i_ the mean bias over n_i_ replicates, using the IQC material level i for the calibrator lot k (or IQC material k, or measuring system k), x_i_ the pooled mean value obtained using the IQC material level i for the calibrator lot k (or IQC material k, or measuring system k), μ_i,k_ the reference value using the IQC material level i for the calibrator lot k (or IQC material k, or measuring system k) - this value can be the value assigned by the manufacturer of the IQC material or conventional value calculated as the mean of arithmetic means of peer-group laboratories participating in an inter-laboratory IQC program (*e.g.* UNITY from Bio-Rad Laboratories) using the IQC material level i for the calibrator lot k (or IQC material k, or measuring system k), 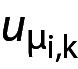 is the uncertainty associated with the reference value μ_i,k_, 
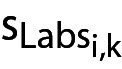
 is the robust peer-group standard deviation obtained using the IQC material level i for the calibrator lot k (or IQC material k, or measuring system k), q_i,k_ the number of peer-group laboratories participating in the IQC material level i for the calibrator lot k (or IQC material k, or measuring system k).

When only one calibrator lot, IQC lot, and a unique measuring system are used during a specified time interval, equations become:


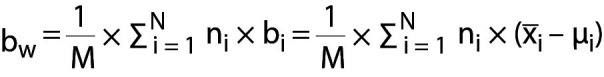



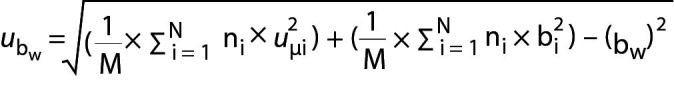


The IQC manufacturer must provide the μi,k. Otherwise, if the IQC material presents an associated IQC inter-laboratory scheme, it can be estimated as (*26*):


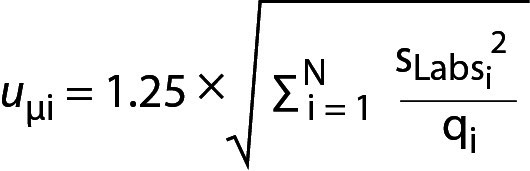


where N represents the number of IQC material levels used, n_i_ the number of replicate measurements using the IQC material level i, M the total number of replicate measurements, b_i_ the bias over n_i_ replicates using the IQC material level i, x_i_ the mean value obtained using the IQC material level i, μ_i_ the reference value for the IQC material level i (this value corresponds to the conventional value calculated as the mean of arithmetic means of peergroup laboratories participating in an inter-laboratory IQC program (*e.g.* UNITY from Bio-Rad Laboratories), *u*_μi_ the uncertainty associated with the mean reference value μ_i_, s_Labsi_ the robust peer-group standard deviation obtained for the IQC material level i and q_i_ the number of peer-group laboratories participating in the IQC material level i.

The bias can also be estimated from EQAS. In these cases, the bias and its uncertainty can be calculated as described above. Thus:

When more than one measuring system is used to measure the same quantity, a mean bias (b) can be calculated as (*24*):


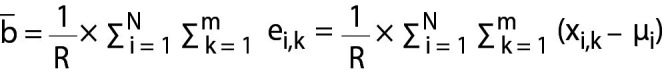


and its uncertainty (*u*_b_) as:


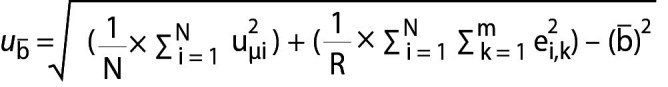


The EQAS’ manufacturer must provide the 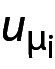 or, if not, could be calculated as (*26*):


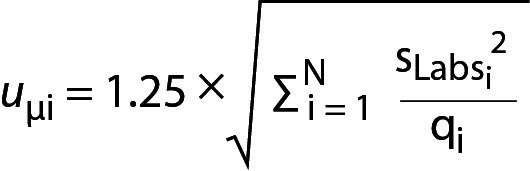


where R is the total pool size (total number of measurements including all measuring systems and EQAS participations, N is the number of EQAS participations, e_i,k_ is the measurement error for the EQAS participation i and the measuring system k, x_i,k_ is the measured value obtained for the EQAS participation i and the measuring system k, μ_i_ is thre reference value assigned by the EQAS manufacturer for the EQAS participation i, 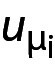 is the uncertainty associated with the reference value μ_i_, 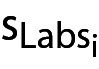 is the robust peer-group standard deviation facilitated by the EQAS manufactured for participation i and q_i_ is the number of peer-group laboratories for the EQAS participation i.

When only one measuring system is used, the bias (b) and its uncertainty (*u*_b_) can be calculated as:


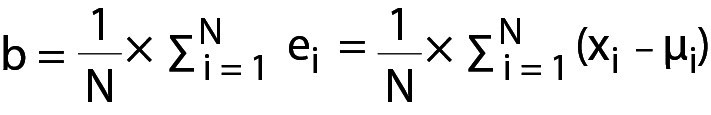



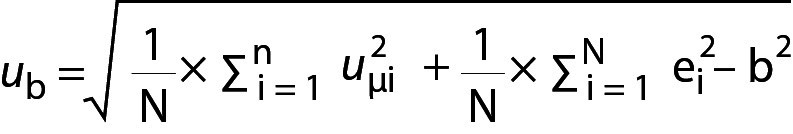



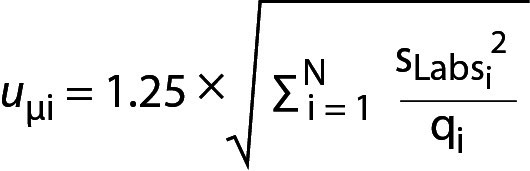


where N represents the number of EQAS participations, e_i_ the measurement error for the EQAS participation i, x_i_ the measured value obtained for the EQAS participation i, μ_i_ the reference value assigned by the EQAS manufacturer for the EQAS participation i, 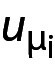 the uncertainty associated with the reference value μ_i_, 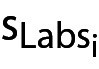 the robust peer-group standard deviation facilitated by the EQAS manufacturer for participation i and q_i_ the number of peer-group laboratories for the EQAS participation i.

Once the bias and its uncertainty have been estimated, metrological compatibility studies can be carried out to know whether the biases are or are not statistically significant. Thus, a bias is considered significant if the absolute value of the bias itself is higher than its relative expanded uncertainty, *i.e.* if |b| > 2 × *u*_b_ (*4*).

If a significant bias is detected, its treatment should be different depending on the kind-of-reference material used. If a CRM is used, the bias should be eliminated by applying a correction factor to every individual measured value obtained, dividing the assigned value of the CRM by the mean value obtained in the bias study. Also, the MU associated with this correction factor (*u*_cf_), calculated such as the *u*_b_, should be included in the uncertainty budget. On the contrary, if IQC or EQAS control materials are used, it is not recommended to apply a correction factor to eliminate the bias, and the bias itself should be included in the uncertainty budget (*6*, *22*, *25*).

### Calculation of the combined standard uncertainty

Wen the individual contribution of each standard uncertainty source has been estimated, the combined standard uncertainty can be calculated by adding estimates of the standard uncertainties considered above, according to one of the following equations:


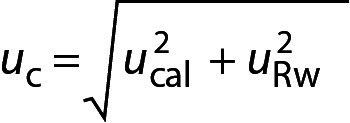



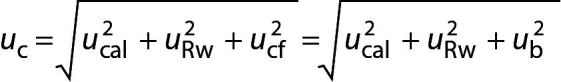



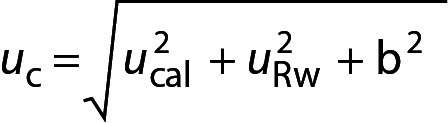


Clinical laboratories should use Eq. 20 when the compatibility study shows that the bias is not statistically significant. Equation 21 should be used when a CRM is used to estimate the bias, the bias is significant, and it has been “eliminated” applying a correction factor. Equation 22 should use if IQC or EQAS materials are used to estimate the bias, and the laboratory cannot “eliminate” the bias.

### Calculation of the expanded uncertainty

Expanded uncertainty (*U*) is calculated multiplying the u_c_ by a coverage factor *k*:

*U* = *k* × *u*_c_ (Eq. 23)

This *k*-value depends on the type of probability distribution, the level of statistical significance selected and the number of independent measurements made to obtain the *u*_c_. Under typical clinical laboratory working conditions, it is acceptable to use a *k*-value of 2 (*6*, *7*).

### Comparison of the expanded uncertainty obtained with the maximum allowable expanded uncertainty

Finally, to know if a *U* value is acceptable, it must be compared with the maximum allowable (permissible) expanded uncertainty (*U*_max_). Thus, an U value is considered acceptable if it is lower or equal than the previously selected *U*_max_ by the laboratory.

Another controversial point that currently exists is how the *U*_max_ should be established. Measurement uncertainty requirements for defining fitness-for-purpose limits may be based on clinical outcome studies, biological variation or state-of-the-art, being those based on biological variation, despite their limitations, generally accepted and used (*27*-*30*). However, it should be noted that unless a country has established legal metrological requirements (*e.g.* the German RiliBÄK), the selection of one type of requirement or another is a matter of consensus and depends on the clinical laboratory itself.

So, despite there are several ways to select the *U*_max_, we show here a procedure based on state-of-the-art to calculate the *U*_max_ using on the RiliBÄK concept named “root mean square of measurement error” (∆) (*31*-*33*):


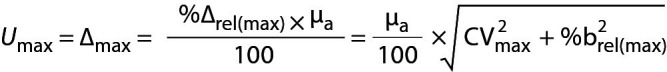


where ∆_max_ represents the maximum allowable absolute root mean square of measurement error, %∆_rel(max)_ the maximum allowable pecent relative root mean square of measurement error, μ_a_ the reference value for which the requirement has been established, CV_max_ the maximum allowable coefficient of variation and %b_rel(max)_ the maximum allowable percent relative bias.

The %∆_rel(max)_ values can be selected directly from RiliBÄK (*31*). Otherwise, they can be calculated from the CV_max_ and %b_rel(max)_ using biological variation data, state-of-the-art data, or data from different organizations such as CLIA, National Cholesterol Education Program for lipid-related quantities, European Medicine Agency (EMA) for drugs, among others (*34*-*38*).

To illustrate the proposal for the estimation of MU, some biological quantities that are routinely measured in clinical laboratories using both already “commercial” (*i.e.*, those with CE marking) and “*in-house*” validated measurement procedures have been selected (see Supplementary material 1). Table 1 shows the MU budget and the maximum allowable relative expanded uncertainty. Besides, Supplementary materials 2, 3 and 4 contain spreadsheets that allow calculating the primary measurement uncertainty sources (*u*_cal_, *u*_Rw_ and *u*_b_), the *u*_c_, and the U. Also, they include a study to know if the *U* obtained is or is not acceptable compared with the *U*_max_, and show an example of how to specify the measurand. Every supplementary material considers the use of the three kind-of-materials to estimate the bias, CRM, IQC materials (with an associated IQC inter-laboratory scheme), and EQAS materials.

**Table 1:** Measurement uncertainty budget for different biological quantities commonly measured in clinical laboratories

**Biochemical quantity**	**Unit**	IQC measured value* (N)	***u*_cal_**	***u*_Rw_^†^**	***b^‡^***	***u*_b_^‡^**	***u*_c_**	***U*** **(*k* = 2)**	***%U*_rel_ (*k* = 2)**	***%U*_rel(max)_**
S-Alanine transaminase; cat.c.	U/L	23.4 (614)	0.5280	1.1523	- 0.1523	1.2347	1.2675	2.5	10.8	11.5
		181 (643)	0.5280	2.3892	- 0.1523	1.2347	2.4468	4.9	3	
S-Albumin; mass c.	g/L	27.1 (639)	0.1490	0.7537	1.2639	0.8140	0.7683	1.5	5.7	12.50
		47.6 (606)	0.1490	1.1801	1.2639	0.8140	1.1895	2.4	5.0	
S-Bilirubin; subst.c.	μmo/L	8.52 (414)	0.5850	0.5314	- 0.5530	1.0540	0.7903	1.58	18.55	22.00 (from 1.7 to < 34 µmol/L) 16.00 (from > 34 to 513 μmol/L)
		107 (410)	0.5850	1.9651	- 0.5530	1.0540	2.0503	4	4	
S-Cholesterol; subst.c.	mmol/L	2.93 (385)	0.0220	0.0527	- 0.0107	0.0621	0.0571	0.11	3.90	4.20
		7.01 (389)	0.0220	0.0842	- 0.0107	0.0621	0.0871	0.17	2.48	
S-Clozapine; subst.c.	nmol/L	618 (131)	16.2486	28.0565	113.6000	96.4195	32.4219	65	11	21.2
		1689 (131)	28.6875	36.8283	113.6000	96.4195	47.2660	95	6	
S-Glucose; subst.c.	mmol/L	3.42 (645)	0.0453	0.0721	0.0159	0.0943	0.0851	0.17	4.98	11.00
		19.1 (632)	0.0453	0.3811	0.0159	0.0943	0.3838	0.7	4.0	
P(aB)-Hydrogen ion; pH (37 °C)	1	7.084 (1723)	0.0071	0.0113	0.0081	0.0599	0.0133	0.027	0.377	0.400
		7.415 (1748)	0.0071	0.0098	0.0081	0.0599	0.0121	0.024	0.326	
		7.622 (1725)	0.0071	0.0094	0.0081	0.0599	0.0118	0.024	0.309	
S-Potassium ion; subst.c.	mmol/L	2.72 (758)	0.0100	0.0414	0.0057	0.0199	0.0426	0.09	3.13	4.50
		4.27 (243)	0.0100	0.0379	0.0057	0.0199	0.0392	0.08	1.84	
		7.89 (783)	0.0250	0.0621	0.0057	0.0199	0.0669	0.13	1.70	
B-Sirolimus; subst.c.	nmol/L	4.31 (210)	0.0492	0.3300	0.6301	0.3122	0.4494	0.90	20.85	21.20
		7.94 (210)	0.0792	0.4838	0.6301	0.3122	0.5753	1.15	14.49	
		12.4 (210)	0.1417	0.6282	0.6301	0.3122	0.7109	1.4	11.5	
S-Thyrotropin; arb.subst.c.	mIU/L	0.74 (333)	0.0100	0.0475	0.2723	0.8914	0.0485	0.01	13.12	13.50
		6.79 (196)	0.0100	0.2731	0.2723	0.8914	0.2733	0.55	8.05	
		36.9 (149)	0.0100	1.7180	0.2723	0.8914	1.7180	3.4	9.3	
IQC - internal quality control. *IQC measured values correspond to the pooled mean values obtained after processing IQC materials for all identical measuring systems used to measure the quantities. *u*_cal_ - uncertainty associated with the values assigned to the calibrator. *u*_Rw_ - uncertainty related to the long-term intermediate imprecision. *b* - measurement bias. *u*_b_ - uncertainty associated with the bias. *u*_c_ - combined standard uncertainty. *U* - expanded uncertainty. *%U*_rel_ - percent relative expanded uncertainty. *k* - coverage factor. *%U*_rel(max)_ - maximum allowable per cent relative expanded uncertainty. Biochemical quantities nomenclature (20): aB - arterial blood; B - blood; P - plasma; S - serum; arb.subst.c. - arbitrary substance concentration; cat.c. - catalytic concentration; mass c. - mass concentration; and subst.c - substance concentration. ^†^To estimate the *u*_Rw_, one calibrator and IQC material lot, and two or more identical measuring systems to measure the selected quantities were used. Instead, for the mass concentration of sirolimus in blood and mass concentration of clozapine in serum, only one calibrator, IQC material lot and measuring system were used. ^‡^The *b* and the *u*_b_ for the substance concentration of sirolimus in blood were estimated using the certified reference material (CRM) ERM-DA111a. This CRM was processed twice per week for six months, and only one measuring system and calibration lot were used. The manufacturer provided the CRM assigned value and its uncertainty, and they were 10.64 nmol/L and 0.301 nmol/L, respectively. The *b* and the *u*_b_ for the mass concentration of clozapine in serum and the pH in arterial blood were estimated using data from 10 external quality assessment scheme participations during the survey period 2019. The *b* and the *u*_b_ for the rest of biological quantities using IQC materials, which they have the inter-laboratory quality control scheme associate it. The IQC data and the conditions used were the same that those described for estimating the *u*_Rw_.										

## Metrological traceability description

As we commented before, the description of MT in clinical laboratories is a less controversy matter than the MU uncertainty and can be made simply based on the ISO 17511:2020 (*5*). All information needed to its description can be provided by the manufacturers of the reagents or calibration materials, as well as from certificates of analysis of CRM declared by international or national metrology institutes, and from the Joint Committee for Traceability in Laboratory Medicine (JCTLM) database (*39*). For each biological quantity, the strategy to follow can be based on:

Obtaining the MT declared by the manufacturers. If this information is not present in brochures or incomplete information is found, this one can be acquired directly asking the manufacturers, or in some cases, from websites of government agencies, such as the Food and Drug Administration (FDA) (*40*).
Obtaining additional information related to the references (units of measurement, measurement procedures, or reference materials) to describe the calibration hierarchies and the sequence of result assignments up the point at which metrological traceability begins. This information can be obtained from the reagent’s manufacturers, CRM certificates, or the JCTLM database (*39*).
Performing a table or flow chart from all information previously collected to describe the metrological traceability chain and the calibration hierarchy of the measurement results.

As an example, Table 2 and Supplementary Material 5 show the MT description for some biological quantities.

**Table 2:** Metrological traceability for different biochemical quantities results usually measured in clinical laboratories

**Biochemical quantity**	**IVD** **manufacturer**	**Calibration-** **material**	**Metrological traceability declared by the manufacturer**		**Highest metrological traceability of quantity results**
			**Reference material**	**Measurement procedures used to prepare/assign their calibration materials**	
S-Alanine transaminase; cat.c.	Roche Diagnostics	Calibrator for automated systems (C.f.a.s) (Ref. 10759350190)	n.d.	IFCC’s method (41)	IFCC/ERM-AD454k *ICRMP: IFCC’s procedure (41) *ICS: ERM-AD454k/IFCC
S-Albumin; mass c.	Roche Diagnostics	Calibrator for automated systems (C.f.a.s) (Ref. 10759350190)	BCR470/CRM470	n.d.	USNRP 12-0575C *ICS: USNRP 12-0575C (42)
S-Bilirubin; subst.c.	Roche Diagnostics	Calibrator for automated systems (C.f.a.s) (Ref. 10759350190)	n.d.	Doumas’ method (43)	Doumas’ procedure *ICRMP: Doumas’ procedure (43)
S-Cholesterol; subst.c.	Roche Diagnostics	Calibrator for automated systems (C.f.a.s) (Ref. 10759350190)	n.d.	Abell-Kendall’s method (44) and ID-MS (45)	ID-GC/MS Abell-Kendall *ICRMP: Abell-Kendall (44) and ID-GC/MS procedures (45)
S-Clozapine; subst.c.	*“In-house”*	*“In-house”*	Clozapine 100 mg [USP] (ref. 1142107)	Gravimetry	Clinical Laboratory of Hospital de Bellvitge’s calibrators *MSMP:* Gravimetry *CRM:* Clozapine, 100 mg [USP] (Ref. 1142107)
S-Glucose; subst.c.	Roche Diagnostics	Calibrator for automated systems (C.f.a.s) (Ref. 10759350190)	n.d.	ID-MS	Roche Diagnostics’s calibrators *MSMP:* ID-MS
P(aB)-Hydrogen ion; pH(37 °C)	Instrumentation Laboratory	GEM Premier 5000 PAK BG/ISE/GL/COOX (ref. 55360011)	SRM 186g	n.d.	SI *PRMP:* Potentiometry using the Harned’s cell without transference and liquid junction *PMS: SRM 186g
S-Potassium ion; subst.c.	Roche Diagnostics	ISE Standard low and high (Ref. 11183974216/ 11183982216)	Primary calibrators (not specified) prepared gravimetrically from purified salts	Gravimetry	SI *PRMP:* Gravimetry and coulometry based on DerSimonian-Laird procedure. Spark-source MS *PMS: SRM 918c
B-Sirolimus; subst.c.	Recipe	ClinCal Whole Blood Calibrator Set, lyophil., for Immunosuppressants (Level 0 – 6) (Ref. 9933)	ERM-AC021a	LC-MS/MS	SI *PRMP:* Gravimetry, HPLC-UV, HPLC-MS, TLC, Karl-Fisher titration, TGA, ^1^H NMR *PMS: ERM-AC021a
S-Thyrotropin; arb.subst.c.	Roche Diagnostics	TSH Calset (Ref. 04738551190)	WHO 3rd IRP 80/558	n.d.	WHO NIBSC 3rd IRP 80/558 *ICS: WHO NISNC 3rd IRP 80/558
CRM - Certified reference material. ERM - European Reference Material. ^1^H NMR - nuclear magnetic resonance of hydrogen 1. HPLC-MS - high-performance liquid chromatography with mass spectrometry detection. HPLC-UV - high-performance liquid chromatography with ultraviolet detection. *ICS* - international conventional standard. *ICRMP* - International conventional reference measurement procedure. ID-MS - isotope dilution-mass spectrometry. ID-GC/MS - isotope dilution-gas chromatography-mass spectrometry. IFCC - International Federation of Clinical Chemistry and Laboratory Medicine. IRP - International Reference Preparation. IVD - *in vitro* diagnostic. LC-MS/MS - liquid chromatography-tandem mass spectrometry. MS - mass spectrometry. *MSMP -* Manufacturer’s selected measurement procedure. n.d. - not declared. NIBSC - National Institute for Biological Standards and Control. *PMS* - primary measurement standard. *PRMP* - primary reference measurement procedure. RM - Reference material. SI - International System of Units. SRM - Standard Reference Material. TGA - thermogravimetric analysis. TLC - thin-layer chromatography. USNRP - United States National Reference Preparation for Human Serum Proteins. USP - United States Pharmacopeia. WHO - World Health Organization. *Included in the JCTLM database (39). Biochemical quantities nomenclature (20): aB - arterial blood; B - blood; P - plasma; S - serum; arb.subst.c. - arbitrary substance concentration; cat.c. - catalytic concentration; mass c. - mass concentration; and subst.c - substance concentration.					

## Conclusions

This review provides practical suggestions of how clinical laboratories could estimate the MU and describe the MT of biological quantities results to help and motivate clinical laboratories to: 1) conduct this type of studies, 2) incorporate information regarding uncertainty and traceability in their reports, and 3) allow them a greater understanding of the importance that these concepts have in the laboratory medicine sciences. Also, in the “clinical laboratory accreditation era”, this review could help laboratories in meeting those ISO 15189 requirements related to these two metrological concepts.
